# The 12-lead ECG in peripartum cardiomyopathy

**DOI:** 10.5830/CVJA-2012-006

**Published:** 2012-07

**Authors:** Kemi Tibazarwa, Bongani Mayosi, Karen Sliwa, Kemi Tibazarwa, Melinda Carrington, Simon Stewart, Karen Sliwa, Geraldine Lee, Melinda Carrington, Simon Stewart

**Affiliations:** Hatter Institute for Cardiovascular Research in Africa, Department of Medicine, Faculty of Health Sciences, University of Cape Town, South Africa; Hatter Institute for Cardiovascular Research in Africa, Department of Medicine, Faculty of Health Sciences, University of Cape Town, South Africa; Hatter Institute for Cardiovascular Research in Africa, Department of Medicine, Faculty of Health Sciences, University of Cape Town, South Africa; Soweto Cardiovascular Research Unit, Chris Hani Baragwanath Hospital, University of the Witwatersrand, Johannesburg, South Africa; Soweto Cardiovascular Research Unit, Chris Hani Baragwanath Hospital, University of the Witwatersrand, Johannesburg, South Africa; Soweto Cardiovascular Research Unit, Chris Hani Baragwanath Hospital, University of the Witwatersrand, Johannesburg, South Africa; Soweto Cardiovascular Research Unit, Chris Hani Baragwanath Hospital, University of the Witwatersrand, Johannesburg, South Africa; Baker IDI Heart and Diabetes Institute, Melbourne, Australia; Baker IDI Heart and Diabetes Institute, Melbourne, Australia; Baker IDI Heart and Diabetes Institute, Melbourne, Australia

**Keywords:** peripartum cardiomyopathy, ECG, baseline, follow up, comparative study, South Africa

## Abstract

**Background:**

The value of the 12-lead electrocardiogram (ECG) to provide prognostic information in the deadly and disabling syndrome peripartum cardiomyopathy (PPCM) is unknown.

**Aims:**

To determine the prevalence of major and minor ECG abnormalities in PPCM patients at the time of diagnosis, and to establish whether there are ECG correlates of persistent left ventricular dysfunction and/or clinical stability at six months of follow up, where available.

**Methods:**

Twelve-lead ECGs were performed at the point of diagnosis on 78 consecutive women presenting with PPCM to two tertiary centres in South Africa and 44 cases (56%) at the six-month follow up. Blinded Minnesota coding identified major ECG abnormalities and minor ECG changes.

**Results:**

The cohort mainly comprised young women of black African ancestry (90%) [mean age 29 ± 7 years and median body mass index 24.3 (IQR: 22.7–27.5) kg/m^2^]. The majority of cases (*n* = 70; 90%) presented in sinus rhythm (mean heart rate 100 ± 21 beats/min). At baseline, at least one ECG abnormality/variant was detected in 96% of cases. Major ECG abnormalities and minor changes were detected in 49% (95% CI: 37–60%) and 62% (95% CI: 51–74%) of cases, respectively; the most common being T-wave changes (59%), p-wave abnormality (29%) and QRS-axis deviation (25%).

Of the 44 cases (56%) reviewed at six months, normalisation of the 12-lead ECG occurred in 25%; the most labile ECG features being heart rate (mean reduction of 27 beats/min; *p* < 0.001) and abnormal QRS axis (36 vs 14%; *p* = 0.014). On an adjusted basis, major T-wave abnormalities on the baseline 12-lead ECG were associated with lower left ventricular ejection fraction (LVEF) at baseline (average of –9%, 95% CI: –1 to –16; *p* = 0.03) and at six months (–12%; 95% CI: –4 to –24; *p* = 0.006). Similarly, baseline ST-segment elevation was also associated with lower LVEF at six months (–25%; 95% CI: –0.7 to –50; *p* = 0.04).

**Conclusions:**

In this unique study, we found that almost all women suffering from PPCM had an ‘abnormal’ 12-lead ECG. Pending more definitive studies, the ECG appears to be a useful adjunctive tool in both screening and prognostication in resource-poor settings.

## Abstract

Peripartum cardiomyopathy (PPCM) is a form of heart failure (HF) with poorly understood aetiology, occurring between the last trimester of pregnancy and up to the first five to six months postpartum.^1^,^2^ Despite an early definition,^3^ later modified by Pearson and colleagues,^4^ there is no consensus regarding PPCM as a single entity among the leading cardiology societies.^5^ The European Society of Cardiology recently declared PPCM a distinct disease entity,^1^ although it may take time before wider awareness of PPCM facilitates more timely diagnosis and pro-active treatment. This is unfortunate given that PPCM causes left ventricular (LV) dysfunction, is more common in particular populations (e.g. African women^6^) and is associated with poor clinical outcomes and survival rates.^7^,^8^

Some studies suggest the incidence of PPCM is one in 3 000 live births. However, one African study found it to be one in 1 000 live births.^9^ There is also a very high risk of relapse in subsequent pregnancies,^10^,^11^ even following full recovery of LV function after the first pregnancy.^6^ Therefore, early and definitive diagnosis of PPCM is essential to limit the high risk of morbidity and mortality in both current and subsequent pregnancies.

Definitive diagnosis and subsequent management of PPCM requires a high index of suspicion. It also usually requires referral to a tertiary centre for echocardiographic studies and specialist cardiological management. Anecdotal evidence suggests that many women who initially present with signs and symptoms indicative of PPCM are diagnosed with ‘non-specific symptoms of the puerperal period’. The misdiagnosis of PPCM (often leading to clinical deterioration and in some instances death) represents a clear target for early intervention and prevention. Until specific aetiologies are identified, PPCM remains a diagnosis of exclusion.

Women in their peripartum period suspected with PPCM require rigorous investigation; a costly and laborious process for the patient and healthcare provider. This is particularly difficult in a resource-poor environment. Although screening with (point-of-care derived) brain natriuretic peptide (BNP) levels may offer a means of detecting elevated atrial pressures secondary to systolic dysfunction (particularly given the age of those affected^6^,^12^,^13^), for example, technical and cost issues remain that prohibit their use. In settings such as sub-Saharan Africa where resources are scarce but the incidence of PPCM is high, the advantages of finding alternative screening tools for this condition that truncate the need for more extensive investigations, while being simple and inexpensive to apply, are abundantly clear.^2^,^14^

Although there is a paucity of electrocardiographic data specifically relating to PPCM, an ‘abnormal’ 12-lead electrocardiogram (ECG) is common in individuals with HF syndrome, with common anomalies including supraventricular arrhythmias, bundle branch block, and sinus bradycardia.^15^ Given the above, we undertook a prospective, pilot study of the 12-lead ECG in a consecutive cohort of newly diagnosed women with PPCM in South Africa. Specifically, the primary aim of this study was to describe the baseline ECG characteristics in PPCM patients, noting the type and prevalence of major and minor ECG abnormalities. We also sought to analyse six-month follow-up ECGs (where available) of PPCM patients to determine potential ECG correlates of persistent LV dysfunction and/or clinical stability, where possible, as repeat ECGs are not part of the routine follow up of PPCM patients.

## Methods

Consecutive patients presenting with *de novo* PPCM to two tertiary centres in South Africa (Chris Hani Baragwanath Hospital, Johannesburg, and Groote Schuur Hospital, Cape Town) between January 2003 and August 2008 were studied. Patients were referred from primary and secondary health facilities, as well as internally from other departments. Only patients aged ≥ 17 years who fulfilled the diagnostic criteria for PPCM4 were considered eligible for the study. For recruitment, previously described^16^ inclusion and exclusion criteria had to be met.

Ethical approval was obtained from each of the local ethical committees of the universities of Cape Town and the Witwatersrand, respectively, prior to the commencement of the study. This study complied with all the requirements of the Declaration of Helsinki. All patients were offered treatment and follow up as per the local standard of tertiary care.

A total of 78 women presenting with PPCM were studied. Of these, 56% had follow-up ECG data and were included in the comparative study analyses. Of those patients who had not had six-month ECGs (*n* = 34), three patients died (3.9%). Importantly, patients with repeat six-month ECG data did not differ significantly with respect to baseline heart rate, NYHA functional class, and left ventricular ejection fraction (LVEF) from the remaining cohort.

All patients with the provisional diagnosis of PPCM underwent a thorough medical interview and examination, and were investigated to confirm the diagnosis at baseline. All patients had a 12-lead ECG and echocardiography. Additional investigations were performed on a case-by-case basis. Data were captured on standardised case report forms.

A 12-lead resting ECG was performed by a trained technician and analysed by a reviewer blinded to all clinical data (GL), using the Minnesota code classification system.^17^ The code allows systematic classification of Q and QS patterns, axis deviation, R waves, ST depression and elevation, T-wave changes, along with conduction abnormalities in both atria and ventricles.^17^,^18^ The abnormalities detected by the Minnesota code were pooled into major abnormalities and minor variations from the ‘normal’ 12-lead ECG using the classification system previously applied by de Bacquer and colleagues^19^ (Table 1). Separate analyses for ST-segment depression, arrhythmia or atrio-ventricular (AV) block, bundle branch block and left-axis deviation were also performed.

**Table 1.** Major Abnormalities And Minor 12-Lead ECG Variations Based On Minnesota Coding

*Major ECG abnormality*	*Minor ECG variations*
Q-wave abnormalities	Borderline Q waves
ST-segment depression	Left- or right-axis deviation
T-wave inversion	High-amplitude R waves
2^o^ or 3^o^ AV block	Borderline ST-segment depression
Complete LBBB or RBBB	T-wave flattening
Frequent premature atrial or ventricular beats	Low QRS voltage
Atrial fibrillation or flutter	

AV = atrio-ventricular; LBBB = left bundle branch block; RBBB = right bundle branch block. (Adapted from de Bacquer *et al.*, 1998).^19^

Standard methods for two-dimensional Doppler transthoracic echocardiography were applied as per the American Society of Echocardiography guidelines.^20^ LV systolic dysfunction was defined by echocardiographic documentation of left ventricular ejection fraction (LVEF) ≤ 45%. All studies were saved onto hard-drive facilities, and a random sample of these was reviewed by a cardiologist blinded to the clinical details of these patients, to confirm the accuracy of parameters describing cardiac structure and function.

## Statistical analyses

All data analyses were performed with STATA-8.^21^ For numerical variables, we report on the mean [standard deviation (SD)] for normally distributed variables, and median [inter-quartile range (IQR)] for non-parametric variables. Comparison between baseline and follow-up ECGs was done using paired *t*-tests for normally distributed numerical variables, Mann-Whitney/Wilcoxon signed rank tests for non-parametric paired numerical variables, and chi-squared tests for categorical variables and proportions. Multivariate analysis was conducted using linear and logistic regression for numerical and categorical outcome variables, respectively.

## Results

**Fig. 1.**
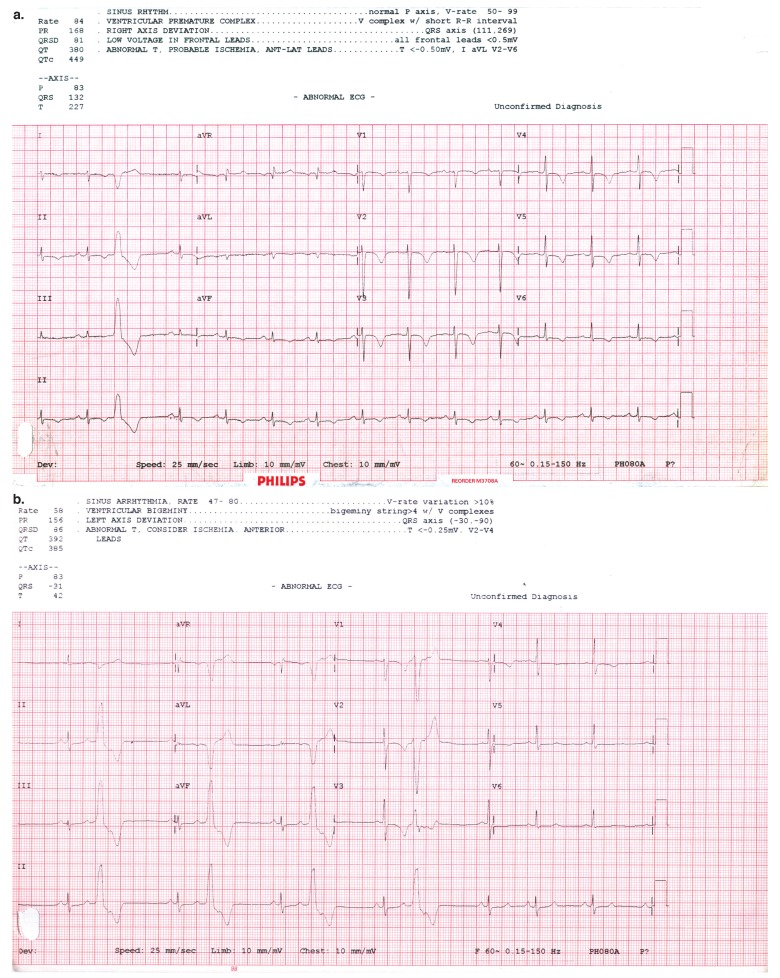
A typical ECG in PPCM at baseline (a) and after six months (b).

Table 2 summarises the clinical and demographic profiles of the 78 women with de novo PPCM, 10 of whom experienced a first-ever detected episode of mildly raised blood pressure at some stage during the index pregnancy. The case report and Fig. 1 describe such a typical case.

**Table 2.** Baseline Clinical And Demographic Profile

Socio-demographic profile	
Mean age (years)	29 ± 7*
Proportion black African (%)	90
Obstetric profile	
Median parity	2 (IQR 1–3)**
Median postpartum period at presentation (days)	18 (IQR 6–30)**
Clinical presentation	
Proportion with New York Heart Association functional class III or IV (%)	64
Median body mass index (kg/m^2^)	24.3 (IQR 22.7–27.5)**
Mean pulse rate	99 ± 19*
Blood pressure (mmHg)	
Mean systolic	116 ± 20*
Mean diastolic	76 ± 14*
2D Doppler echocardiography	
Median intra-ventricular septal thickness	0.9 (IQR 0.8–1.1)**
in diastole (cm)	5.8 ± 0.7*
Mean left ventricular end-diastolic diameter (cm)	30.5 ± 9*
Mean ejection fraction (%)	

*Standard deviation (± SD); **interquartile range (IQR).

Interestingly, no patients under the age of 17 years presented to either study unit and 90% of patients included were young women of African ancestry. Of the 10% that were of non-black African ethnicity, almost all were of mixed ancestry, with only one Caucasian patient. The majority of respondents were normotensive and experienced onset of symptoms in the postpartum period (median 18 days, IQR 6–30 days). However, 8% of respondents reported the onset of symptoms prepartum, of which only two were hypertensive (one mild and the other with moderate hypertension, defined as per standard protocol).^22^-^24^

**Fig. 2.**
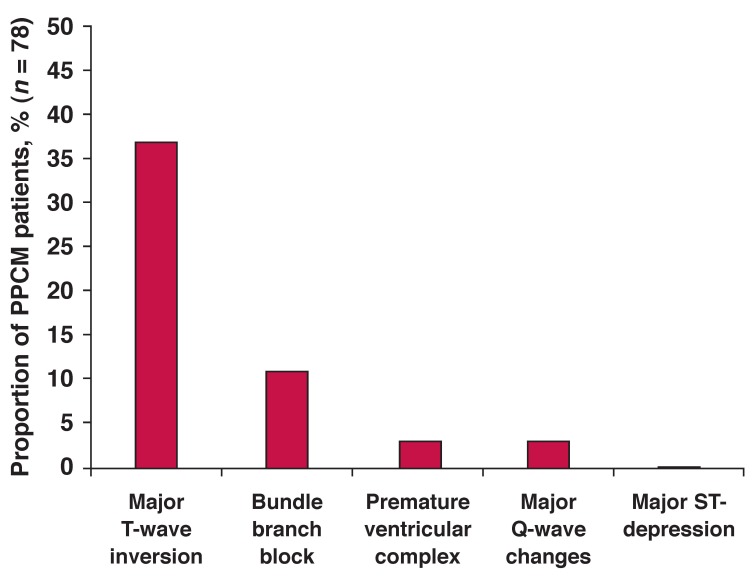
Prevalence of Minnesota major criteria at baseline in 78 PPCM patients.

Table 3 summarises baseline ECG abnormalities/variations from normal (*n* = 78). The majority of cases (90%) were in sinus rhythm, although mean heart rate was markedly elevated, with 45% of cases in sinus tachycardia (defined as those ≥ 100 beats/min, given that our patients’ maximum heart rate was 134 beats/min). Only three patients (4%) had completely normal ECGs; excluding those with an elevated heart rate, this increased to nine patients (12%). Overall, 49% (95% CI: 37–60) of cases had a major Minnesota ECG abnormality detected, while 62% (95% CI: 51–74) had a minor ECG variant. A combined total of 63 (81%; 95% CI: 70–89%) cases had one or both forms of abnormality detected on their 12-lead ECG. Of the major abnormalities, major T-wave anomalies (38%), followed by abnormal QRS axis (26%) were the most common (Fig. 2). T-wave anomalies were also the most common of all documented ECG abnormalities overall (59%), followed by atrial abnormalities (29%).

**Table 3.** 12-Lead ECG At Baseline In 78 Ppcm Patients

	*ECG characteristic*	*No. (%) (n = 78)*
Rate and rhythm	Mean heart rate (beats/min) ± SD	100 ± 21
	Proportion in sinus rhythm	70 (90%; 95% CI: 81–95)
	Proportion with sinus tachycardia	35 (45%; 95% CI: 34–57)
	Proportion with arrhythmias	
	• premature ventricular complex	3 (4%; 95% CI: 0.8–11)
	• supraventricular tachycardia	1 (1%; 95% CI: 0.03–7)
	• sinus arrhythmias	4 (5%; 95% CI: 1–13)
Axis	QRS axis	
	• abnormal	20 (26%; 95% CI: 16–37)
	• left axis	9 (12%; 95% CI: 5–21)
	• right axis	8 (10%; 95% CI: 5–19)
	• indeterminate	3 (4%; 95% CI: 0.8–11)
Conduction	PR interval > 220 ms	1 (1%; 95% CI: 0.04–7)
	Proportion with bundle branch block (BBB)	9 (12%; 95% CI: 5–21)*
	• left BBB	4 (5%; 95% CI: 1–13)
	• right BBB	1 (1%; 95% CI: 0.03–7)
	Proportion with prolonged QTc (> 470 ms)	4 (5%; 95% CI 1–13)
Repolarisation	Proportion with T-wave abnormalities	46 (59%; 95% CI: 47–70)**
	• major	30 (38%; 95% CI: 28–50)
	• minor	24 (31%; 95% CI: 21–42)
	Proportion with ST-segment changes	
	• major ST-segment changes	1 (1%; 95% CI: 0.03–7)
	• minor ST-segment changes	3 (4%; 95% CI: 0.8–11)
	• ST-segment elevation	1 (1%; 95% CI: 0.03–7)
Hypertrophy	Proportion with left ventricular hypertrophy [Defined by Minnesota codes III_1_ and (IV_1-3_ or V_1-3_)]	7 (9%; 95% CI: 4–18)
Atria	Proportion with atrial abnormalities	23 (29%; 95% CI: 20–41)**
	• left atrium	8 (10%; 95% CI: 5–19)
	• right atrium	11 (14%; 95% CI: 7–24)
	• bi-atrial	4 (5%; 95% CI: 1–13)

*This sum exceeds that of individual BBB as some patients manifested incomplete BBB (either left or right). **This sum exceeds that of individual sub-categories as some patients manifested features of each sub-category.

Univariate analysis showed no association between LV systolic function and baseline ECG readings. However, on adjustment for age, functional class, echocardiographic LV dimensions, and all the other ECG parameters listed in Table 3, major T-wave abnormalities correlated negatively with left ventricular systolic dysfunction. The presence of major T-wave changes was associated with a clinically relevant 9% (95% CI: 1–16; *p* = 0.03%) reduction in LVEF compared to those without T-wave changes.

At six months, a number of clinical parameters had improved in surviving cases subjected to study follow up (*n* = 44) (Table 4). Overall, 55% had no residual evidence of LV systolic dysfunction (*p* < 0.001), although 10% still reported functional impairment (NYHA class II or more). Overall, 25% of this sub-set of cases had a normal 12-lead ECG at six months.

**Table 4.** Comparing Baseline And Six-Month ECG Characteristics In 44 PPCM Patients With Follow-Up Data

		*Baseline % of population*	*6-month follow up % of population*	p*-value*
Rate and rhythm	Mean heart rate (beats/min) ± SD	104 ± 18	77 ± 14	< 0.001
	Proportion in sinus rhythm	84 (95% CI: 70–93)	66 (95% CI: 50–80)	0.049
	Proportion with sinus tachycardia	48 (95% CI: 32–63)	7 (95% CI: 1–19)	< 0.001
	Proportion with arrhythmias			
	• premature ventricular complex	2 (95% CI: 0.06–12)	2 (95% CI: 0.06–12)	1.0
	• sinus arrhythmias	7 (95% CI: 1–19)	27(95% CI: 15–43)	0.011
Axis	Proportion with QRS axis being:			
	• abnormal	36 (95% CI: 22–52)	14(95% CI: 5–27)	0.014
	• left axis	18 (95% CI: 8–33)	7 (95% CI: 1–19)	0.107
	• right axis	11 (95% CI: 4–25)	5 (95% CI: 0.6–15)	0.237
	• indeterminate	7 (95% CI: 1–19 )	2 (95% CI: 0.06–12)	0.306
Conduction	Proportion with bundle branch block (BBB)	20 (95% CI: 10–35)*	18 (95% CI: 8–33)	0.787
	• left BBB	9 (95% CI: 3–22)	9 (95% CI: 3–22)	1.0
	• right BBB	2 (95% CI: 0.06–12)	2 (95% CI: 0.06–12)	1.0
Repolarisation	Proportion with T-wave abnormalities	45 (95% CI: 30–61)**	27(95% CI: 15–43)	0.123
	• major	30 (95% CI: 17–45)	34 (95% CI: 20–50)	0.647
	• minor	16 (95% CI: 7–30)	9 (95% CI: 3–22)	0.334
	Proportion with ST-segment changes			
	• major ST changes	0	0	–
	• minor ST changes	2 (95% CI: 0.06–12)	0	0.315
	• ST-segment elevation	2 (95% CI: 0.06–12)	0	0.315
Hypertrophy	Proportion with left ventricular hypertrophy [Defined by Minnesota Codes III_1_ and (IV_1-3_ or V_1-3_)]	7 (95% CI: 1–19 )	7 (95% CI: 1–19 )	1.0
Atria	Proportion with atrial abnormalities	20 (95% CI: 10–35)**	9 (95% CI: 3–22)	0.133
	• left atrium	5 (95% CI: 0.6–15)	2 (95% CI: 0.06–12)	0.557
	• right atrium	9 (95% CI: 3–22)	7 (95% CI: 1–19)	0.694
	• bi-atrial	7 (95% CI: 1–19 )	0	0.078

*This sum exceeds that of individual BBB as some patients manifested incomplete BBB (either left or right). **This sum exceeds that of individual sub-categories as some patients manifested features of each sub-category.

According to univariate analyses, no difference between ‘clinically stable/responded to treatment’ (i.e. recovered LV function) versus non-responders (persistent LV dysfunction) were found in respect of any ECG parameter. However, on an adjusted basis, major T-wave changes and major ST-segment depression found on the baseline 12-lead ECG subsequently correlated with persistently impaired systolic function at six months. Specifically, the presence of major T-wave changes at baseline was associated with a markedly lower LVEF at six months (–12%, 95% CI: –4 to –21; *p* = 0.006) compared to those without T-wave changes at baseline. In addition, ST-segment elevation at baseline was associated with an even greater reduction in LVEF at six months (–25%, 95% CI: –0.7 to –50; *p* = 0.044) compared to those without this ECG pattern at baseline.

## Discussion

To the best of our knowledge, this is the first study to systematically describe the 12-lead ECG in *de novo* cases of PPCM. Our main aim was to examine the potential utility of the 12-lead ECG (a relatively inexpensive and easy-to-apply diagnostic tool) in detecting underlying LV dysfunction in confirmed cases of *de novo* PPCM in African women. This would require a high underlying level of ECG abnormalities in such a cohort in order to discriminate against (presumably) more normal 12-lead ECGs in African women experiencing healthier pregnancies.

Of the 78 cases studied, 49% demonstrated major ECG abnormalities, usually associated with significant underlying cardiac pathology, while 62% demonstrated one or more forms of minor variation/abnormality, potentially indicative of the same. We also attempted to examine whether the 12-lead ECG is a useful tool for discriminating between those cases who respond to treatment (by the resolution of initially observed ECG abnormalities) and those who had persistent LV dysfunction. In this respect, we found that the presence of two major abnormalities (T-wave inversion and ST-segment depression) and a third Minnesota code criterion not listed as one of the major or minor criteria (ST-segment elevation) found on the baseline 12-lead ECG correlated with persistently poor LV systolic function at six months. T-wave inversion also correlated with LV systolic function at baseline.

Typically, LV systolic functional recovery in PPCM is a slow and drawn-out process that enters the second year of treatment.^2^ On this basis, while LVEF in those patients subjected to six-month follow up improved overall, just under half still had defined impaired LV dysfunction, and this represents a major therapeutic target for treatment. Therefore, long-term follow up using the ECG in PPCM might well show ECG reversal to normality as late as 18 months after first diagnosis, as our long-term echocardiographic data suggest.^8^ Moreover, we have identified potentially useful markers (i.e. major T-wave inversion and/or ST-segment depression on the 12-lead ECG) as simple but important prognostic markers that might trigger more intensive/aggressive treatment and follow up in PPCM cases.

Our findings and the overall utility of the 12-lead ECG in this clinical setting require careful interpretation when fundamental investigations such as echocardiography remain inaccessible to most hospitals and patients in sub-Saharan Africa. Serum levels of NT-proBNP are known to strongly predict the degree of heart failure,^12^ yet this test is still not available in most referral hospitals in Africa where PPCM is prevalent. Surprisingly, because of vast differences in sensitivity and specificity in detecting HF, it has been suggested that the overall cost-effectiveness of measuring serum NT-proBNP becomes comparable to that of screening for HF using the 12-lead ECG alone,^25^,^26^ due mainly to the relatively low specificity of the 12-lead ECG.^26^

The scarcity of the serum NT-proBNP test in our setting almost mandates using something as inexpensive and easy as the 12-lead ECG to screen for PPCM, even if its sensitivity and specificity prove to be imperfect. These data will be particularly useful if (after comparing ECG patterns in healthy African women, derived from the Heart of Soweto cohort^27^) the 12-lead ECG has the potential to be applied as a ‘rule-out’ test (i.e. high specificity to identify all truly negative for PPCM cases). Unfortunately, the ability to combine 12-lead ECG with typical symptoms of HF (to increase its accuracy in detecting PPCM) is confounded by their parallel presence in the late stages of pregnancy (but not typically post-partum).

As indicated, our data suggest that baseline major T-wave abnormalities were associated with poorer LV systolic function at baseline, and, alongside baseline ST-segment depression, they were also associated with persistent LV systolic dysfunction in the short to medium term (i.e. six months). In Western countries, major ST-segment depression and T-wave abnormalities are often regarded as indications of myocardial ischaemia, bearing consistent prognostic significance for cardiovascular disease mortality across prospective studies, especially for men.^28^

We remain wary of the fact that gender differences in ECG findings often show women to have a higher prevalence of ST-segment depression or T-wave changes, such as to question the true significance of any association between ST-segment depression and T-wave abnormalities with coronary heart disease (CHD) mortality in women.^28^ However, we are greatly reassured by the number of large, population-based studies that show major ST-depression to be the most predictive ECG characteristic of cardiovascular disease (CVD) and CHD mortality, lending an average two-fold risk of CVD and CHD mortality, and, as with our study, predicting these outcomes from their mere presence at baseline.^19^

Studies reporting on the ECG in prognostication of PPCM remain scarce, with two from Nigeria suggesting it to be a weak predictor of recovery and long-term prognosis in PPCM.^29^,^30^ However these studies did not use echocardiography to confirm the diagnosis of PPCM,^30^ and had a greater proportion of patients with hypertension than those without.^29^,^30^ Given recent insight that patients with the PPCM phenotype who present with hypertension appear to follow a different natural history to those without,^2^ any comparisons between data from PPCM patients with hypertension with those without hypertension should be interpreted with great caution.

Systematic reports of ECG abnormalities associated with idiopathic dilated cardiomyopathy (IDCM), which bears some resemblance to PPCM, are few. One such study of IDCM reported that the ECG was found to be normal in up to 25% of affected relatives of IDCM patients (who by definition suffer from familial DCM), although these were not all newly diagnosed IDCM cases.^31^ Hence, as in our study, an overwhelming majority had abnormal ECGs.

It remains fair to say that the shortage of studies systematically reporting on the prevalence of ECG anomalies in PPCM may account for most for our inability to corroborate our findings with other available evidence, yet no reports contradict our findings. Overall, therefore, our data can only suggest that the 12-lead ECG in PPCM may be sensitive to underlying LV dysfunction and/or that it can serve as a marker of more extensive cardiac insult early in the disease process.

Lastly, we note that the 1% prevalence of first-degree heart block, usually not considered to bear any significant risk to adverse CVD outcome, except for its association with lamin A/C mutation in familial DCM, may in our PPCM cohort merely reflect the 1–2% prevalence in normal young adults.^32^ However, after recent reports implicating this so-called benign form of heart block in the general population with increased risk of atrial fibrillation and adverse CVD outcome 20 years down the line,^32^ and suggestions that post-exercise measurements of PR intervals may be more prognostic within five-year follow-up periods than resting ECG PR intervals,^32^ it would be useful to revise the prognostic implications of first-degree heart block post exercise in patients with underlying myocardial disease and congestive cardiac failure as in PPCM.

Furthermore, evaluation of this conduction disorder may be of particular importance in PPCM, in view of several reports of first-degree AV block being among one of the earliest signs of lamin A/C mutation, which in turn commonly leads to a phenotype of apparently unexplained DCM.^33^ It is worth considering that the prognostic implications behind each of the criteria for major and minor ECG abnormalities/variants derived from the Minnesota code appear more applicable in the screening of high-risk persons only,^34^ as in this clinical context.

This pilot study has a number of limitations that require comment. Firstly, the Minnesota code may not be sufficiently validated for the detection of heart disease in pregnant women. Moreover, the ‘normal’ 12-lead ECG in African women is yet to be definitively described. Whether the combined presence (81% of cases) of major abnormalities and minor variations is sufficient to support further investigation of the 12-lead ECG as a screening tool (particularly when there are few data to describe the ‘normal’ 12-lead ECG in pregnant and non-pregnant African women), is open to debate.

In determining the sensitivity of ECG changes over time, relative to underlying LV dysfunction, we had valid data for only 56% of the cohort. Although our PPCM patients who did not have follow-up ECGs did not differ in clinical and echocardiographic outcome from those with follow-up data, the possibility that the former group may have been clinically worse off than those with six-month data cannot be excluded, given that greater proportions of the former manifested minor T-wave anomalies, and that their left ventricular diameters and BMI at baseline appeared greater than those who had six-month ECGs. However, in light of the lower prevalence of QRS-axis deviation and bundle branch block among the former group, interpretation of the similarity of patients with and without follow up with regard to ECG characteristics becomes speculative.

Further data are required to better characterise the 12-lead ECG as a marker of LV dysfunction in this specific clinical (and ethnic) context, before any firm recommendations can be made in respect of obtaining a 12-lead ECG at the conclusion of each pregnancy in sub-Saharan Africa.

## Conclusions

Despite a number of limitations, this still represents a unique study that will prove to be invaluable in determining the future role of the 12-lead ECG as an inexpensive and simple ‘rule-out’ screening tool for PPCM, and perhaps an important tool for increasing the intensity of subsequent treatment and management. Overall, we found the majority (96%) of PPCM patients presented with ‘abnormal’ 12-lead ECGs, which improved significantly to 75% after the first six months of treatment. Over 80% of patients displayed either major abnormalities or minor variations using the Minnesota code. Of these, sinus tachycardia and QRS-axis deviation were most likely to be attenuated after six months. Even though these ECG abnormalities were mostly non-specific and similar to those of other dilated cardiomyopathies, our study further suggests the ECG to be useful in simple monitoring of clinical progress during treatment and prognostication.

Specifically, the baseline presence of major T-wave and ST-segment abnormalities in the context of PPCM patients may place these patients at similar risk of adverse outcomes to those with myocardial ischaemia. More definitive studies are required to determine if this simple and relatively inexpensive tool will prove to be of particular clinical use in the setting of PPCM. Any progress in this regard will be welcome, given the persistently poor health outcomes associated with PPCM in resource-poor settings.
